# A Shortest Dependency Path Based Convolutional Neural Network for Protein-Protein Relation Extraction

**DOI:** 10.1155/2016/8479587

**Published:** 2016-07-14

**Authors:** Lei Hua, Chanqin Quan

**Affiliations:** ^1^Department of Computer and Information Sciences, Hefei University of Technology, Hefei 230009, China; ^2^Department of Computer and Information Sciences, Kobe University, Kobe 6578501, Japan

## Abstract

The state-of-the-art methods for protein-protein interaction (PPI) extraction are primarily based on kernel methods, and their performances strongly depend on the handcraft features. In this paper, we tackle PPI extraction by using convolutional neural networks (CNN) and propose a shortest dependency path based CNN (sdpCNN) model. The proposed method (1) only takes the sdp and word embedding as input and (2) could avoid bias from feature selection by using CNN. We performed experiments on standard Aimed and BioInfer datasets, and the experimental results demonstrated that our approach outperformed state-of-the-art kernel based methods. In particular, by tracking the sdpCNN model, we find that sdpCNN could extract key features automatically and it is verified that pretrained word embedding is crucial in PPI task.

## 1. Introduction

Biomedical relations play an important role in biologic processes and are widely researched in the field of biomedical natural language processing (BioNLP). PPI task aims to extract protein interactions; for example, in sentence* “The distribution of actin filaments is altered by profilin overexpression,”* the interaction between protein entities* “actin”* and* “profilin”* would be extracted. A number of databases, such as BIND [1], MINT [2], and IntAct [3], had been created to store structured interactions. However, the biomedical literature regarding protein interactions is expanding rapidly, making it difficult for these databases to keep up with the latest protein-protein interactions. Consequently, effective and automatic protein-protein relation extraction systems become more significant.

Previous researches have illustrated the effectiveness of the shortest dependency path (sdp) between entities for relation extraction in many fields [4–7]. For example, in PPI task, [8] proposed an edit-distance kernel based on sdp and classified the relations by SVM. Reference [9] has made a detailed investigation into the relevant work of relation extraction and elaborated the important role of sdp in relation extraction. However, how to preprocess the sdp (e.g., using a variety of kernels) and how to combine different features (e.g., part-of-speech, *n*-grams, and parser tree) still are open problems. In this work, the proposed approach takes raw sdp as the only input, and it can learn features automatically. And thus, different from previous researches, manual feature selection and feature combination are not necessary in our approach.

Many efforts have been done on PPI task, especially the kernel based methods. Most of these methods take the PPI task as a binary classification problem by determining whether there is an interaction between the two entities. The kernels include bag-of-words kernel [10], all-path kernel [11], subset-tree kernel [12], edit-distance kernel [8], and graph kernel [13], and they have shown effectiveness in PPI task. Considering that single kernel partly calculates the similarity of two instances, hybrid kernel [14–17] has been proposed and demonstrated much better performance than single kernel. Kernel methods are effective, because they integrate a large amount of manually selected features. The problem of existing kernel based method is how to combine different features; in most cases, sophisticated design is required.

Deep learning methods have achieved remarkable results in computer vision [18] and speech recognition [19], and due to much of the effective work involved in neural network language models (NNLM) [20, 21], recently, some work has focused on neural network especially CNN for natural language processing (NLP) problem. Using CNN to extract features for NLP was previously researched by the authors in [22]; they considered the tasks including part-of-speech (POS), chunking, name entity recognition (NER), and semantic role labeling (SRL) as sequential labeling problems. In recent years, researches have proposed the use of CNN to extract features for relation extraction. Reference [23] combined the word representation, lexical level features, and word features and used the CNN model to learn the sentence-level features; the features were then concatenated into a vector and fed to a Softmax layer to classify the relationship. Reference [24] shared a similar idea to [23]; the authors proposed a new logistic loss function and a pairwise method to train their CNN model.

However, the CNN based methods described by [23, 24] usually take whole sentence or the context between two target entities as input. The problem of these methods is that such representations fail to describe the relationships of two target entities far in sentence distance, and the irrelevant information may also be considered due to the long distance. Considering the described problems and the complexity of PPI task, in this work, we use dependency parsing to analyze the sentence for generating the sdp at first to capture semantical and syntactical features and then send sdp to sdpCNN for classification.

Comparing with the prior work, the contributions of our work can be concluded as follows:

1. We propose a new model (sdpCNN) to tackle PPI task and show that sdpCNN model built on word embedding is effective in extracting protein-protein relations.
2. We demonstrate that sdpCNN with pretrained word embedding performs much better than randomly generated word embedding and state-of-the-art kernel based methods. It could be concluded that the well pretrained word embedding is important in PPI task.
3. The proposed model is able to extract key features automatically such that the manual feature selection procedure can be avoided.

## 2. Material and Methods

In this section, we firstly introduce word embedding, and then we describe the proposed sdpCNN model in detail. The proposed model consists of three parts: the sdp extraction, sdpCNN based feature extraction, and multilayer perceptron (MLP) based classification.

### 2.1. Introduction for Word Embedding

**Figure 1:**
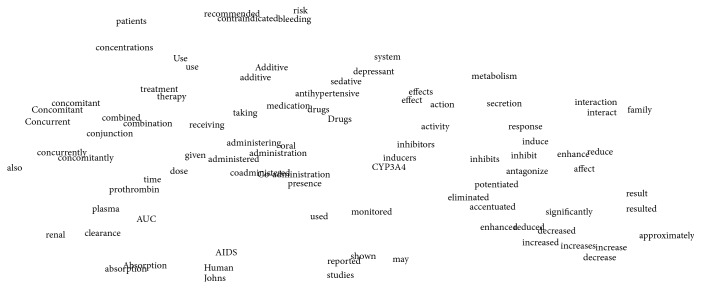
Visualization of word embedding by t-SNE. The words are highly frequent in PPI task. The original word embedding for each word is a 300-dimension vector; all of these words are reduced to 2 dimensions by t-SNE.

Word embedding is a feature learning technique in NLP where words or phrases from the vocabulary are mapped to vectors of real numbers in a low-dimensional space relative to the vocabulary size. Many methods have been proposed to train the word embedding, but most of the methods are based on the distributional hypothesis: words that occur in similar contexts tend to have similar meanings. Given this hypothesis, the trained word embeddings would be close to each other in vector space when the words contain similar meanings (Figure 1 shows visualization of word embedding by t-SNE [25]).

In this work, we use public available pretrained word embedding (300-dimension), trained on 100 billion words from Google News by word2vec [21] (https://code.google.com/archive/p/word2vec/), to build the proposed sdpCNN model.

Compared with traditional* “one-hot”* representation, pretrained word embedding brings about three advantages. (1) It could capture semantic information and weaken word gap problem; for example, in Figure 1, interaction verbs (interaction verbs usually indicate the relation among entities and thus they are important in PPI task)* “affects”* and* “enhance”* are clustered together; however, in traditional* “one-hot”* representation, the verbs* “affects”* and* “enhance”* are completely different. (2) Data sparseness problem could be avoided since all words are mapped into low-dimensional vectors. (3) Pretrained word embedding is trained on large unlabeled corpora, and thus it could enlarge the coverage of vocabulary and decrease the number of unknown words.

### 2.2. Shortest Dependency Path (sdp) Extraction

**Figure 2:**
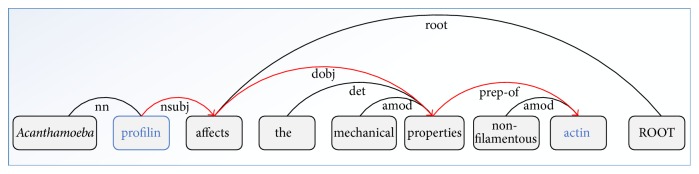
The dependency parsing result of sentence “*Acanthamoeba profilin affects the mechanical properties of non-filamentous actin.*” The words in blue are the two target proteins, and the sdp between the proteins is represented by the red arrows. Tags such as “*nsubj*” and “*dobj*” are the dependency relations between two words.

Semantic dependency parsing had been frequently used to dissect sentence and to capture word semantic information close in context but far in sentence distance. To extract the relationship between two entities, the most direct approach is to use sdp. The motivation of using sdp is based on the observation that the sdp between entities usually contains necessary information to identify their relationship [9]. For example, in Figure 2, the word* “affects”* in sdp provides useful information for classifying two target proteins, and the dependency relationship such as “*nsubj*” (the dependency relation “*nsubj*” represents “*nominal subject*,” and the governor of this relation is always a verb, because interaction verbs are crucial in PPI task; thus, this dependency relation is important in PPI task; more detailed descriptions for relation “*nsubj*” can be found in [26]) between words “*profilin*” and “*affects*” also adds supplemental information for classification.

To reduce the sparseness and ensure the generalization of features, we replace two target proteins with special symbols “*Protein1*” and “*Protein2*,” respectively, and thus we can get a sdp* “Protein1-nsubj-affects-dobj-properties-prep-of-Protein2”* from Figure 2.

### 2.3. sdpCNN Model for Feature Extraction

**Figure 3:**
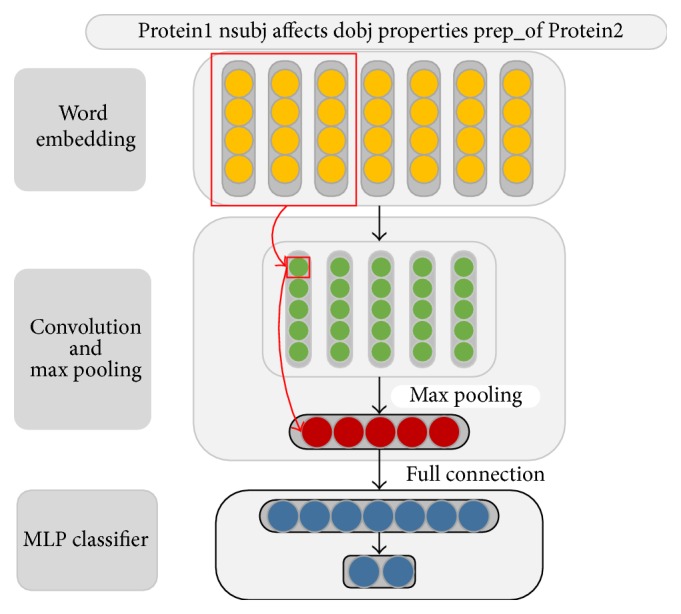
The framework of sdpCNN model with 3-word window. In this example, the input sdp has 7 words (dependency relations such as “*nsubj*” and “*dobj*” are also considered as words), each word embedding is 4 dimensions, and 5 filters are used. The yellow part is the matrix representation for an input sdp; each column in the green part represents the feature map generated by a filter through (1) and (2); and the red part represents the max-pooling results by taking the maximum value over each column in the green part by (3). The arrows in red show the process of generating a feature map *c* ^*∗*^. The blue part is a MLP classifier with a full-connection layer and a Softmax layer.

Figure 3 shows the architecture of the proposed sdpCNN model. In the first step, the model transforms a sdp into a matrix representation by looking up pretrained word embedding; and then, a convolution layer is applied to this matrix to automatically extract the features. The following max-pooling operation generates the most useful local features. At last, the extracted features are fed to a multilayer perceptron (MLP) with a hidden layer and a Softmax classifier.

For notation, we use **D** ∈ *R* ^|*V*|×*d*^ to represent pretrained word embedding, where *V* is the vocabulary of corpora and *d* is the dimension of word embedding. Suppose **x** = {*x*_1_, *x*_2_, *x*_3_,…, *x*_*N*_} is an input sdp with length *N* (we fix the length of input path as *N* by truncating or padding special symbol* “PADDING”*). When we assign each word in sdp **x** with a corresponding row vector from **D**, we would get a matrix representation **P** ∈ *R* ^*N*×*d*^ for input sdp (yellow part in Figure 3).

The convolutional operation would be considered to apply filter **W** ∈ *R* ^*h*×*d*^ to the *h*-word window in input sdp **x**. An *h*-word window in input sdp can be represented as **P**_*i*,*i*+*h*−1_ ∈ *R* ^*h*×*d*^ (yellow part surrounded with red rectangle in Figure 3) by connecting row *i* to *i* + *h* − 1 in **P**. A feature *c*_*i*_ can be generated by

(1) $$\begin{array}{c} c_{i}=f\left(\;W⊙P_{i,i+h-1}+b_{1}\;\right), \end{array}$$

where *f* is an activation function such as hyperbolic tangent (tanh), *b*_1_ is the bias term, and ⊙ is element-wise multiplication. By applying filter to each word window of the input sdp, the model will produce a new feature which we call feature map **c** in

(2) $$\begin{array}{c} c=\left[\;c_{1},c_{2},\ldots ,c_{N-h+1}\;\right]. \end{array}$$

Max-pooling operation (see (3)) takes the maximum value over all the word windows in feature map **c** which brings about two advantages: (1) it could extract the most important local features and (2) it reduces the computational complexity by reducing the feature dimension. Hence,

(3) $$\begin{array}{c} c^{*}=\max\left(\;c\;\right). \end{array}$$

As each filter produces a feature *c* ^*∗*^, multiple filters will generate multiple features. Suppose *M* is the number of the filters; the model would get fixed-size distributed features **r** = [*c*_1_^*∗*^, *c*_2_^*∗*^,…, *c*_*M*_^*∗*^], where *c*_*i*_^*∗*^ is the *i*th feature generated by *i*th filter.

### 2.4. MLP for Classification

A MLP model is employed to calculate the probability of each class. Given the distributed representation **r**, the full-connection weight matrix **W**_2_ ∈ *R* ^*H*×*M*^, the number of hidden layers *H*, and the bias term *b*_2_, the output of full-connection layer **O** ∈ *R* ^*H*×1^ is calculated by

(4) $$\begin{array}{c} O=f\left(\;W_{2}r+b_{2}\;\right). \end{array}$$

Before applying Softmax layer for classification, the original feature space is transformed into confidence space. The input for Softmax layer **I** ∈ *R* ^*C*×1^ is described by

(5) $$\begin{array}{c} I=W_{3}O, \end{array}$$

where **W**_3_ ∈ *R* ^*C*×*H*^ is a transformation matrix and *C* is the number of classes. This task is binary classification, so *C* is 2.

Each value in **I** represents the confidence of the current sample that belongs to each class. A Softmax layer normalizes the confidence to [0,1]. Given **I** = [*i*_1_, *i*_2_,…, *i*_*C*_], the output of Softmax layer **S** = [*s*_1_, *s*_2_,…, *s*_*C*_]. The Softmax operation can be calculated by (6). Both *s*_*j*_ and *p*(*j*∣**x**) represent probability of sdp **x** that belongs to class *j*. Hence,

(6) $$\begin{array}{c} s_{j}=p\left(\;j\;|\;x\;\right)=\frac{e^{i_{j}}}{\sum_{k=1}^{C}e^{i_{k}}}. \end{array}$$

### 2.5. Training Procedure

There are several parameters that need to be updated during the training: the multifilter **W**, the full-connection weight **W**_2_, the transformation matrix **W**_3_, and the bias terms *b*_1_ and *b*_2_. All of the parameters are represented by *θ* = (**W**, **W**_2_, **W**_3_, *b*_1_, *b*_2_). We apply Negative Log-Likelihood (NLL) in (7) (**y**_*i*_ ∈ {0,1} is annotated label for the input sdp **x**_*i*_) as loss function. In order to minimize the loss function, we use gradient descent (GD) based method to learn the network parameters. For each input pair (**x**_*i*_, **y**_*i*_), we calculate the gradient (using the chain rules) of each parameter relative to loss and update each parameter with learning rate *λ* by (8). It is notable that fixed learning rate *λ* would lead to unstable loss in training. In this work, we use an improved GD based algorithm, Adadelta [27], to update the parameters in each training step. Adadelta is able to dynamically adjust the learning rate. Hence,

(7) loss=−log⁡pyi ∣ xi,

(8) θ=θ−λ∂ lossθ.

## 3. Results

### 3.1. Experimental Setup

#### 3.1.1. Datasets

Two standard datasets (both datasets are available at http://corpora.informatik.hu-berlin.de/), Aimed and BioInfer [28], are used to evaluate our model. Aimed was manually tagged by [9] which included about 200 medical abstracts with around 1900 sentences and was considered as a standard dataset for PPI task. BioInfer was developed by Turku BioNLP group (see details at http://bionlp.utu.fi/) which contained about 1100 sentences. If there is an interaction between the two entities, we consider this instance as a positive one; otherwise, we consider it as a negative one (in Table 1). Text preprocessing includes sentence splitting, word segmentation, and dependency parsing (Stanford parser was utilized).

#### 3.1.2. Word Embedding Initialization

In experiments, we compare the performances of pretrained embedding with randomly initialized word embedding. When the words that appeared in the datasets are not included in the pretrained word embedding, we follow [29] and initialize word embedding by randomly sampling from [−*a*, *a*], where *a* is the variance of pretrained word embedding trained by word2vec. For random part, all of the words are initialized by sampling from [−*a*, *a*].

#### 3.1.3. Model Hyperparameters Settings

We experimentally choose the hyperparameters for the model on BioInfer and Aimed datasets shown in Table 2. The Discussion gives details on parameter selection as well as the impact of the parameters.

#### 3.1.4. Evaluation Metrics

We use precision (*P*), recall (*R*), and *F*-score (*F*) to evaluate the performances of our sdpCNN model. *F* is the harmonic mean of recall and precision which is defined by (9). 10-cross-validation (10-fold CV) method is used to calculate the average *F*-scores. Hence,

(9) $$\begin{array}{c} F=\frac{2\times P\times R}{P+R}. \end{array}$$

### 3.2. Performance Comparison

We evaluate our system and compare the performance with state-of-the-art kernel based methods. We start from a baseline model with randomly initialized word embedding, and then we evaluate our model with the pretrained word embedding.  Table 3 shows the comparison results in detail.

We firstly compare the performance with other sdp based methods, and then we compare the results with hybrid kernels based methods. The descriptions for methods in Table 3 are as follows:

 
*Walk-Weighted Subsequence Kernel* [30]. Generating sdp at first and then integrating the proposed e-walk and v-walk kernels for classification.
 
*Graph Kernel* [13]. Encoding the dependency parser results into a graph, proposing an all-path graph kernel by leveraging sdp; at last, least squares support vector machine is used for classification.
 
*SDP-CPT* [4]. Using both sdp and directed constituent parser tree for classification.
 
*Tree Kernel* [6]. On the bias of SDP-CPT, considering the modal verb phrases and appositive dependency features.
 
*Edit-Distance Kernel* [8]. A semisupervised machine learning approach (TSVM) with edit-distance kernel based on sdp.
 
*Hybrid Kernel* [14]. A combination of bag-of-words (BOW) kernel, subset-tree (ST) kernel, and graph kernel.
 
*Multiple Features and Parser* [31]. A combination of rich features including bag-of-words features, sdp features, and graph features.
 
*Multiple Kernel* [32]. A weighted multiple kernel by combining parser tree, graph features, POS, and sdp.

As we can see, kernel methods listed in Table 3 usually require sophisticated design and complex feature combination, and feature engineering still accounts for a large proportion of these systems. In this work, we avoid manual features selection and features combination by using CNN. In addition, the features used in these kernel based methods are all discrete; therefore, the* “word gap”* problem is inevitable, while, by leveraging word embedding and CNN, we can train our model in continuous space and avoid hard assignment.

The main differences of the sdp based methods listed in Table 3 are how sdps were used and how similarity functions were calculated. For example, the most direct way is to encode sdp into* “one-hot”* representation and use SVM for classification [4, 6]. Another way is by using edit-distance kernel [8] to calculate the similarity of two sdps through Levenshtein distance. Compared with these sdp based methods in Table 3, even the baseline model achieved competitive results. Furthermore, pretrained sdpCNN model improved the *F*-scores by 12.4 and 6.4 compared with tree kernel [6] and edit-distance kernel [8] on BioInfer and Aimed datasets, respectively.

It has been verified that a combination of multiple kernels could improve the effectiveness of kernel based PPI extraction methods. Kernels such as tree kernel, graph kernel, and bag-of-words kernel are commonly used in hybrid kernel based methods. Compared with the methods listed in Table 3, the baseline model alone yielded competitive results and improved the *F*-scores by 5.3 on BioInfer dataset when compared with [14]. By integrating pretrained word embedding, our pretrained sdpCNN model exceeded 7.1 and 1.6 compared with [14, 32] on BioInfer and Aimed datasets in Table 3. The experimental results showed that, with the appropriate expression (the sdp in this work) of the relationship, the sdpCNN model built on word embedding can get much better results than the combination of a variety of features (or kernels).

For better understanding extracted features by sdpCNN, Figure 3 illustrates the way of generating a feature map *c* ^*∗*^ in sdpCNN model. By following the negative direction of the red arrows in Figure 3, we can find which word window contributes most to the final classifier. Considering the example in Figure 3, the 3-word window (*“Proteins nsubj affects”*) circled with a red rectangle is key item. We define the word in the middle of the key word window as key-word, and thus the word* “nsubj”* in the middle of the 3-word window* “Proteins nsubj affects”* in Figure 3 is key-word. Each filter produces a key-word; consequently, *M* filters will generate *M* key-words. In our experiments, we noticed that interaction verbs such as* “inhibits*,*” “cause*,*”* and* “bind”* were often chosen as key-words by sdpCNN model. Generally, the construction of an interaction verbs dictionary manually requires a great deal of time and effort, but our model can extract these verbs automatically.

Moreover, the experimental results also showed that the proposed method achieved considerably higher precision (73.4 on BioInfer dataset and 64.8 on Aimed dataset) than the existing approaches.

### 3.3. Evaluation on Different Scales of Training Data

**Figure 4:**
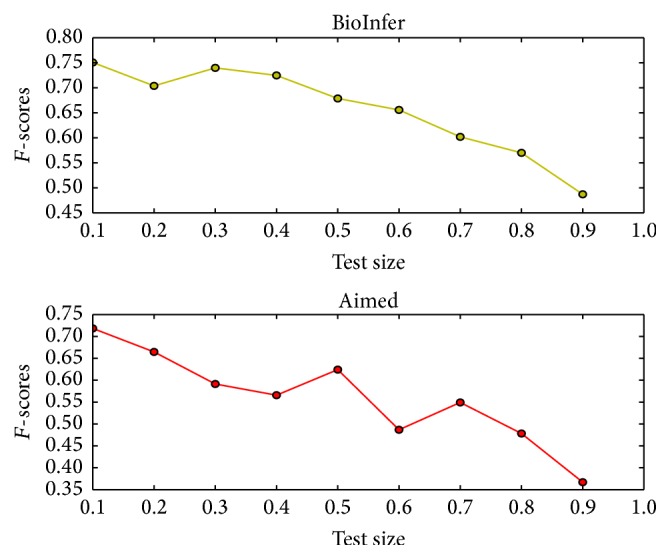
Changes of performance on different scales of training data. The *x*-axis represents the proportion of test data, and the *y*-axis corresponds to *F*-scores. The pretrained sdpCNN model is used in this experiment.

In order to investigate the effect of different scales of training data, we split the original datasets by different ratios.  Figure 4 shows the changes of performance on different scales of training data. As we can see, the performance varied significantly depending on the size of training and test corpus, and *F*-scores changed from 75.1 to 48.2 on BioInfer dataset and 71.1 to 36.2 on Aimed dataset when proportion of test data ranged from 0.1 to 0.9; too few training data would have the risk of loss of data information; as a result, the trained sdpCNN model cannot well generalize the original data which would lead to poor performance.

### 3.4. Discussion

In this section, we firstly investigate the impact of hyperparameters and provide general parameters settings for sdpCNN. After that, we compare the performances among the four proposed methods in Table 5. At last, we manually analyze the errors of sdpCNN alone with the possible solutions to errors.

#### 3.4.1. The Influence of Different Hyperparameters Settings

Consider the following:

(1) Window size *h*: a 3-word window is commonly used in many related works [22–24]; we tested a 2-word window on both Aimed and BioInfer datasets. On Aimed dataset, the results remained essentially unchanged; however, when tested on BioInfer dataset, *F*-scores reduced by 5. We also tested a 4-word window, while, in this experiment, performances are markedly inferior on both datasets, which means a 4-word window is too long to capture the structure information.
(2) The length of fixed-size sdp *N*: the lengths of most paths (more than 95%) in Aimed dataset are less than 20, while, in BioInfer dataset, most of the path lengths (more than 95%) are less than 30. And thus we set *N* with 20 and 30 on Aimed and BioInfer datasets, respectively.
(3) The filters size *M*: due to the limited size of corpora, when the filters size is too big, the model is prone to overfitting; we heuristically choose *M* as 100 in our experiments.
(4) The number of full-connection layer units *H*: based on the idea of [33], the appropriate increment of full-connection layer units could improve the performance. But too many units also suffer from overfitting, so we set *H* with 500 in this experiment.

#### 3.4.2. Comparisons among the Four Proposed Models

*Random sdpCNN Model versus Pretrained sdpCNN Model*. From Table 3, we can find that the pretrained sdpCNN model performed much better than random sdpCNN model and improved the *F*-scores by 1.8 and 3.3 on BioInfer and Aimed datasets, respectively. Intuitively, the pretrained word embedding could capture the semantic information of words, which means words with similar semantics are clustered together in the vector space (Figure 1).  Table 4 shows the examples of neighboring words of target words based on cosine similarity; we can see that word, for example,* “affect*,*”* shares a similar meaning with words “*impacting*,” “*jeopardize*,” and so forth. However, when we randomly allocated the word embedding, semantic information among words would be discarded; as a result, random sdpCNN model might correctly classify the sentence “*Protein1 affects Protein2*” but fails on the sentence “*Protein1 impacts Protein2*” although both sentences indicate interactions. Random sdpCNN model is somewhat similar to the “*one-hot*” model; the trained random sdpCNN model can be well applied to the test data only when train and test instances contain common words which means this model is too dependent on cooccurrence of words and lacks good generalization ability. However, as a benefit from sdp and CNN, the structure information could be well preserved; therefore, random sdpCNN model still achieved comparable results. More specifically, it could be concluded that pretrained sdpCNN model can capture both semantic information and structural information, while the random sdpCNN model could only keep structural information. Both semantic information and structural information play important roles in PPI task.

*Random sdpCNN Model versus Random (Update) sdpCNN Model*. In random (update) sdpCNN model, we considered word embedding as hyperparameters and updated it in the training procedure. The experimental results showed that the random (update) sdpCNN model had a slight improvement (0.7 and 0.6 *F*-scores improvements on BioInfer and Aimed datasets, resp.) compared with the random sdpCNN model. Intuitively, the random (update) sdpCNN model can adapt to the specific task by fine-tuning word embedding which means word embedding can learn task specific patterns. However, when compared with pretrained sdpCNN model, the model's performances reduced by 1.1 and 2.7 on BioInfer and Aimed datasets. The good performance on pretrained sdpCNN model is understandable due to the fact that the pretrained word embedding is trained on large corpora which ensures that the pretrained sdpCNN model could obtain abundant semantic information. Moreover, because the pretrained sdpCNN model does not need to update word embedding, the training time consumption could be reduced. 

*Combined Model versus Pretrained sdpCNN Model*. To better learn the representation of the raw sdp input, we also proposed a model that combined the pretrained and random word embedding (see details in Table 5). The combined model improved the *F*-scores by 0.6 on Aimed corpus and kept the performance on BioInfer corpus when compared with pretrained sdpCNN model. However, it is also notable that the combined model would take more than two times the cost on training time. There is always a trade-off between time and performance.

Among these four models, pretrained sdpCNN model is more time-saving (relative to combined model and random (update) sdpCNN model), robust (relative to random (update) sdpCNN model), and effective (relative to random (update) sdpCNN model and random sdpCNN model). In conclusion, a CNN model built on high-quality pretrained word embedding could be considered as an effective alternative in PPI task.

#### 3.4.3. Errors Analysis

Confined to the complexity and diversity of the biomedical expressions, extracting relations from biological articles remains a big challenge. In this subsection, we carefully analyze the errors of sdpCNN and list the three typical errors as follows:

1. When an input sentence is too long, the Stanford dependency analysis tool is prone to errors, and because our model is built on sdp the propagation of errors would lead to poor performance of sdpCNN.
2. When irrelevant interaction verbs are included in sdp, as mentioned before, interaction verbs strongly suggest interactions; as a result, the model would make a mistake.
3. Randomly initialized word embedding would also hurt the system's performance. In our system, the dependency relations such as “*nsubj*” and “*prep-of*” are all considered as input words, and such words are not likely to be included in pretrained word embedding, and thus these words are randomly assigned with vectors. As a result,* “nsubj”* and* “prep-of”* might be far from each other in vector space. For example, for two input paths* “Protein1-nsubj-bind-nsubj-Protein2”* and* “Protein1-nsubj-bind-prep-of-Protein2*,*”* both paths indicate interactions; however, the sdpCNN model could only distinguish the first one.

The possible solutions for the mentioned errors are described as follows: the first error could be weakened by integrating the context between two target entities, because the context could provide supplementary information when standard tools fail to capture dependency relations among words. As for the second error, a possible solution is to introduce position information, because, in most of the time, the relevant interaction verbs locate in the middle of two target entities. For randomly initialized word embedding problem, we might take word embedding as hyperparameter and update it during the training. Meanwhile, word embedding used in this work is trained on large unlabeled Google News; it would be better to train word embedding on large biological articles to enrich semantic information.

## 4. Conclusion

In this paper, we have described a sdpCNN model built on word embedding for PPI task. Experiments demonstrated that our method outperformed the state-of-the-art kernel based methods. The main contribution of the proposed method is the integration of word embedding, sdp, and CNN. Word embedding is able to capture semantic information and effectively weaken word gap problem. By applying sdp and CNN, the proposed model could make full use of structure information and avoid manual feature selection. Our experimental results also indicated that (1) the raw sdp input is crucial to describe protein-protein relationship in PPI task; (2) the CNN model is useful to capture the local features and structure information; (3) high-quality pretrained word embedding is important in PPI task. Through error analysis, we notice that there still is room for improvement. In our future work, we would like to train our own word embedding and design our PPI system by making full use of context information, position information, and sdp.

## Figures and Tables

**Table 1:** Data statistics for Aimed and BioInfer datasets.

Datasets	Positive	Negative
BioInfer	2512	7010
Aimed	995	4812

**Table 2:** Hyperparameter settings for Aimed and BioInfer.

Datasets	*N*	*d*	*h*	*M*	*H*
BioInfer	30	300	3	100	500
Aimed	20	300	3	100	500

**Table 3:** The comparison with other kernel based methods on PPI task. *Random sdpCNN model*: sdpCNN model with randomly initialized word embedding. *Pretrained sdpCNN model*: sdpCNN model built on pretrained word embedding.

Method	BioInfer			Aimed		
	*P*	*R*	*F*	*P*	*R*	*F*
Random sdpCNN model (baseline)	69.6	77.8	73.4	54.5	75.2	62.7
Pretrained sdpCNN model	**73.4**	77.0	**75.2**	**64.8**	67.8	**66.0**

sdp based methods						
Walk-weighted subsequence kernel	61.8	54.2	57.6	61.4	53.3	56.6
Graph kernel	—	—	—	52.9	61.8	56.4
SDP-CPT	—	—	62.4	—	—	58.1
Tree kernel	—	—	62.8	—	—	51.4
Edit-distance kernel	—	—	—	58.4	61.2	59.6

Hybrid kernel based methods						
Hybrid kernel	65.7	71.1	68.1	55.0	68.8	60.8
Multiple features and parser	—	—	67.6	—	—	64.2
Multiple kernel	57.0	77.3	65.8	57.7	71.1	64.4

**Table 4:** The top 5 neighboring words of target words based on cosine similarity (the variants of the target words, such as “*induced*,” “*inducing*,” and “*depended*,” are not included in this table).

Target words	1	2	3	4	5
Induce	*elicit*	*suppress*	*provoke*	*potentiate*	*engender*
Affect	*impacting*	*jeopardize*	*hinder*	*impair*	*imperil*
Bind	*vise*	*attach*	*untie*	*glue*	*entangle*
Depend	*rely*	*hinge*	*predicated*	*affect*	*dictate*
Prevent	*deter*	*avoid*	*discourage*	*forestall*	*avert*

**Table 5:** The results of the four proposed models on PPI task. *Combined model*: using both randomly initialized and pretrained word embedding as inputs and concatenating the outputs of max-pooling layer as features for MLP. *Random (update) sdpCNN model*: initializing word embedding randomly and updating word embedding during training.

Method	BioInfer	Aimed
	*F*	*F*
Combined model	**75.3**	**66.6**
Pretrained sdpCNN model	75.2	66.0
Random sdpCNN model	73.4	62.7
Random (update) sdpCNN model	74.1	63.3
